# Feasibility of tele-guided patient-administered lung ultrasound in heart failure

**DOI:** 10.1186/s13089-023-00305-w

**Published:** 2023-02-09

**Authors:** Ariella Pratzer, Eugene Yuriditsky, Tajinderpal Saraon, Michael Janjigian, Ali Hafiz, Jun-Chieh J. Tsay, Pamela Boodram, Nikita Jejurikar, Harald Sauthoff

**Affiliations:** 1 NYU Grossman School of Medicine, 550 First Ave, New York, NY USA; 2 VA New York Harbor Healthcare System, 423E 23rd Street, New York, NY USA

**Keywords:** Lung ultrasound, Tele-guidance, Patient-performed, Heart failure

## Abstract

**Background:**

Readmission rates for heart failure remain high, and affordable technology for early detection of heart failure decompensation in the home environment is needed. Lung ultrasound has been shown to be a sensitive tool to detect pulmonary congestion due to heart failure, and monitoring patients in their home environment with lung ultrasound could help to prevent hospital admissions. The aim of this project was to investigate whether patient-performed tele-guided ultrasound in the home environment using an ultraportable device is feasible.Affiliations: Journal instruction requires a country for affiliations; however, these are missing in affiliations [1, 2]. Please verify if the provided country are correct and amend if necessary.Correct

**Methods:**

Stable ambulatory patients with heart failure received a handheld ultrasound probe connected to a smart phone or tablet. Instructions for setup were given in person during a clinic visit or over the phone. During each ultrasound session, patients obtained six ultrasound clips from the anterior and lateral chest with verbal and visual tele-guidance from an ultrasound trained clinician. Patients also reported their weight and degree of dyspnea, graded on a 5-point scale. Two independent reviewers graded the ultrasound clips based on the visibility of the pleural line and A or B lines.

**Results:**

Eight stable heart failure patients each performed 10–12 lung ultrasound examinations at home under remote guidance within a 1-month period. There were no major technical difficulties. A total of 89 ultrasound sessions resulted in 534 clips of which 88% (reviewer 1) and 84% (reviewer 2) were interpretable. 91% of ultrasound sessions produced interpretable clips bilaterally from the lateral chest area, which is most sensitive for the detection of pulmonary congestion. The average time to complete an ultrasound session was 5 min with even shorter recording times for the last session. All patients were clinically stable during the study period and no false positive B-lines were observed.

**Conclusions:**

In this feasibility study, patients were able to produce interpretable lung ultrasound exams in more than 90% of remotely supervised sessions in their home environment. Larger studies are needed to determine whether remotely guided lung ultrasound could be useful to detect heart failure decompensation early in the home environment.

## Introduction

Acute decompensated heart failure (HF) results in an excess of 1 million hospital admissions per year in the United States with approximately 25% of patients being readmitted within 30 days [1–3]. The economic burden of HF readmissions is substantial; the average HF-related admission costs in excess of $14,000, with over half of the $39.2 billion of direct costs of HF care attributable to hospital treatment [2, 4]. Most HF admissions are the result of progressive rise in intracardiac filling pressures leading to pulmonary congestion, which is characterized by an increase in extravascular lung water [5].Abbreviations: In Abbreviations section, the term “ICD” was repeated twice. So we have remove the repeated one. Kindly check and confirm.confirmed

Unfortunately, clinical features and cardiac biomarkers have limitations in detecting early manifestations of HF, and as a result, there is considerable interest in remote monitoring technologies [6, 7] that can detect HF decompensation at an early stage. Implantable hemodynamic monitoring systems, such as the CardioMEMS device, which are placed in the distal pulmonary artery, can detect early increases in pulmonary arterial pressure and have been shown to significantly decrease HF hospitalizations by up to 37% over a 15-month period [2, 3, 7]. However, this technology is invasive, and the cost of the device and implantation exceeds $19,000, with 5-year costs exceeding $188,000 per CardioMEMS patient [8].

Lung ultrasound (LUS), an easy to use, non-invasive, and affordable technology, is an appealing alternative to current options for early detection of HF, and has shown promise in its applicability to this field. B-line artifacts, laser-like vertical hyperechoic reverberations arising from the pleural line, are indicative of extravascular lung water [9–12]. B-lines observed with LUS correlate significantly with intracardiac filling pressures obtained by right heart catheterization and with radiographic pulmonary edema [9–14]. Although the presence of B-lines is not specific for pulmonary congestion, the absence of B-lines is highly predictive (> 90%) for low/normal pulmonary artery occlusion pressures [11]. Multiple studies have found LUS to be significantly more predictive of the diagnosis of HF than either biomarkers or clinical assessment, in settings spanning the pre-hospital, emergency, and inpatient setting [15–18].

In contrast to echocardiography, LUS image acquisition is simple, and windows require far less precision. Even in non-expert hands, LUS has very good accuracy in evaluating cardiogenic causes of dyspnea [9, 19–21]. Multiple studies have demonstrated strong agreement in LUS interpretation between experts and novices [15, 20, 21]. Short training sessions allow nurses to perform and interpret LUS in the evaluation of HF with high predictive value [19].

Recently, ultra-portable, low-cost ultrasound devices with remote guidance capability have become available, opening up the possibility of various tele medicine applications. The aim of this study was to explore the feasibility of tele-guided patient-administered lung ultrasound to detect early manifestations of HF. An FDA-approved, portable, hand-held ultrasound system consisting of a probe connected to a smart phone or tablet was distributed to patients with stable HF. Patients were remotely guided through image acquisition and uploading, and the recorded clips were analyzed for image quality and diagnostic usability.

## Methods

### Study design and population

Stable, ambulatory patients with NYHA Class I–III HF from the NYU Langone Heart Failure clinic were recruited for the study. Patients were eligible for inclusion if they were 18 or older and had NYHA class I–III HF. Patients were deemed ineligible if they were unable or refused to participate in the initial training session, or if they were unable to demonstrate successful image acquisition and probe manipulation at the conclusion of the training session. Patients were consented for this study during an in-person HF clinic visit or via tele visit with mailing of the consent form. This study was approved by the New York University Langone Health Institutional Review Board, and appropriate guidelines were followed.

Each patient was provided a Butterfly iQ handheld ultrasound probe, along with a charger, a compatible smart phone or tablet (patients 1 to 3), a phone charger, a phone or tablet holder, and set up instructions. The first 3 patients were instructed on how to use the probe during an in-person clinic visit, and were equipped with the probe and a tablet at that time. During the height of the pandemic, the study was initially paused, but was then restarted using an entirely remote approach. All items were mailed to the patient, and instructions on set up and probe usage were provided via video call. Each patient performed 10–12 guided remote ultrasound sessions within a 1-month period.

**Fig. 1:**
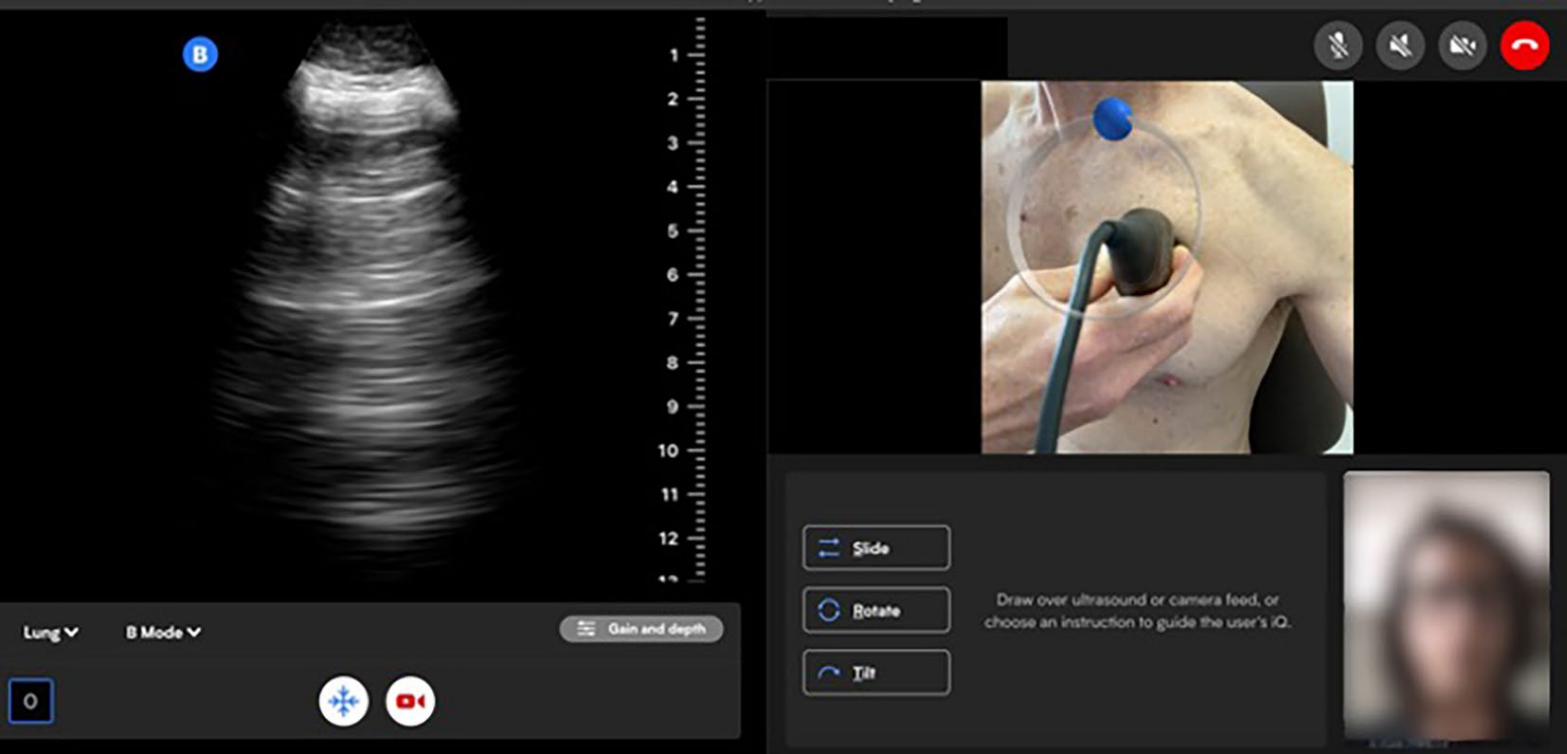
Example of a patient-performed remotely guided lung ultrasound session from the viewpoint of the guiding clinician

During each ultrasound session, patients obtained and transmitted six ultrasound clips under tele-guidance with an ultrasound trained clinician. Guidance was provided both verbally and visually, with the clinician able to draw on the patient’s chest via video (Fig. 1). Three clips for each side of the chest were obtained with the patient in a semi-recumbent position. Previously, in patients with HF, the 3rd intercostal space in the anterior and mid axillary line was found to have the highest sensitivity for the detection of B-lines [22]. Based on this study, we selected these locations in addition to the mid clavicular line, also in the 3rd intercostal space.

During each tele-guidance session, patients were also asked to report a 5-point Likert dyspnea scale score and their daily weight. Ultrasound clips were then uploaded and stored in a secure online network archive, without any personal identifying information. The de-identified clips were later reviewed and evaluated by two independent reviewers. Clips were assessed for visibility of the pleural line/rib interspace and visibility of at least one A-line or any B-lines to be graded as interpretable. Gain and depth were also assessed and graded as either appropriate or inappropriate. Depth settings of at least 13 cm were graded as appropriate. As gain settings cannot be objectively quantified, this was left to the judgement of the reviewer.

### Statistical analyses

Outcomes were analyzed at the individual patient level. The primary outcomes were percentage of interpretable clips and number of lung ultrasound exams with at least four out of six interpretable clips. Inter-reviewer agreement was also analyzed using Cohen’s Kappa.

## Results

Eight stable ambulatory heart failure patients were recruited for this feasibility phase of the study (Table 1). Patient age ranged from 30 to 77 years, with mean age of 52 years. Six (75%) of the patients were male. Five (62.5%) of the patients were white, three (37.5%) were black, and two (25%) were Hispanic/Latino. Five (62.5%) of the patients had an implantable cardioverter defibrillator (ICD) in place, and ejection fraction (EF) ranged from 17% to 75%, with an average EF of 34.6%. Dry weight ranged from 52 to 160 kg, with an average dry weight of 106.3 kg.

**Table 1:** Patient characteristics

Characteristic	Value
Age range	30–77 years
Age median	53
Male	6 (75%)
Female	2 (25%)
Black	3 (37.5%)
White	5 (62.5%)
Hispanic/Latino	2 (25%)
Ejection fraction (average)	17–75% (34.6%)
ICD in place	5 (62.5%)
Dry weight range	52–160 kg
Dry weight average	106.3 kg

**Fig. 2:**
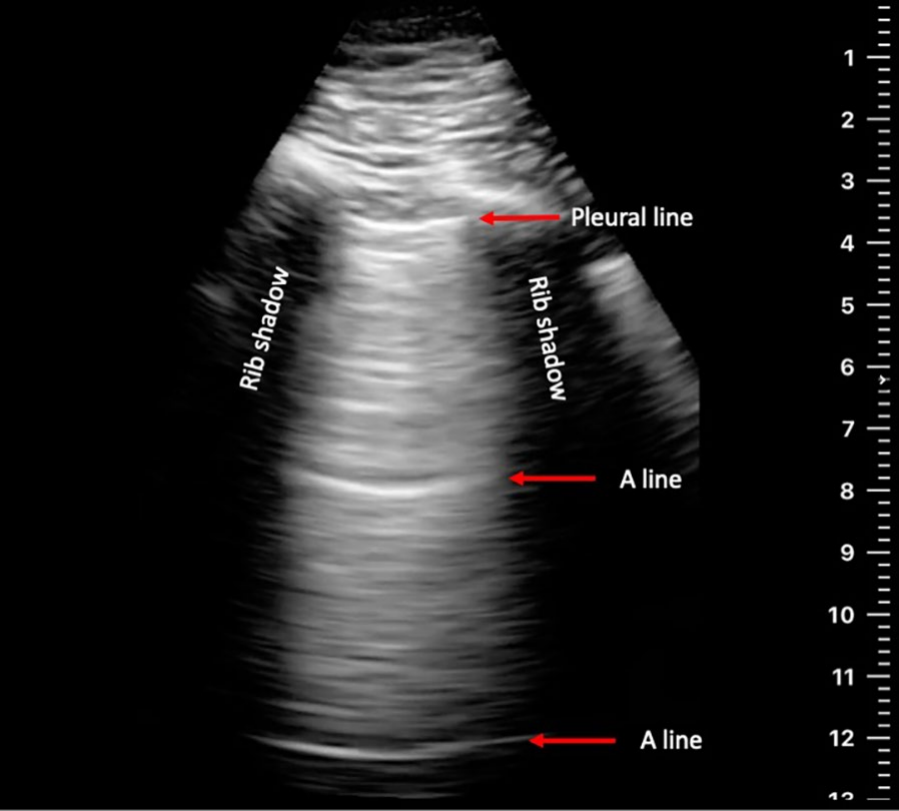
Representative image of a patient-performed ultrasound clip

Patients performed a cumulative total of 89 guided remote ultrasound sessions. From those 89 sessions, a total of 534 independent LUS clips were obtained and analyzed. A representative clip is shown in Fig. 2. There were no major technical issues. Two independent reviewers viewed and graded all collected ultrasound clips. 88% of all clips were interpretable per reviewer one, and 84% per reviewer two. 92.1% of LUS exams had at least 4 out of 6 interpretable clips per reviewer one, and 89.9% per reviewer two. Inter-reviewer agreement was 93%, with a Cohen’s Kappa of 0.7. More than 97% of all clips were determined to have appropriate gain and depth settings.

As mentioned above, the sensitivity for the detection of B-lines in HF patients is highest in the lateral lung zones [22, 23]. The LUS locations with the highest diagnostic accuracy for HF in this study were obtained in the mid axillary line in the third intercostal space. Although lateral LUS is harder for patients to self-perform, 75.3% of the remote ultrasound sessions produced interpretable mid axillary LUS clips bilaterally. If broadened to include the anterior axillary line, which is nearly as sensitive in the diagnosis of heart failure [22], 91.0% of the remote ultrasound sessions produced interpretable lateral clips bilaterally.

The average time to obtain all six ultrasound clips to complete a remote ultrasound session was 5.04 min. This time was calculated from the time of capture of the first clip to the time of capture of the last clip for each session, and, therefore, does not include the time to adjust the probe for the first recording. All eight patients had a shorter average time for their last three sessions than for their first three sessions, with an average time of 3.38 min for the last session, suggesting improvement in ease of use over time.

Two patients continued performing LUS without supervision, after the conclusion of their participation in the study, and transmitted their images for review. For these 13 unsupervised sessions, 100% of LUS clips were graded as interpretable.

Four of the eight patients had an implanted CardioMEMS device and had PAP recordings on the same days as their remote ultrasound sessions. While these data can only be looked at qualitatively given the small number of patients, fluctuations in PAP did not clearly track with dyspnea scores or with changes in daily weight. It is worth noting that the average dry weight for patients in this study was 106.3 kg, and that weight did not compromise the quality of the LUS clips.

Finally, in this patient group with stable HF, no abnormal B-lines were found during this study, and none of the enrolled patients were admitted to the hospital for a HF exacerbation. The highest Likert dyspnea score recorded was a three out of five, and six of the eight patients reported dyspnea scores of one throughout the study. None of the patients gained a significant amount of weight during the study. Hence, there were no clinical HF exacerbation, and no false positive B-lines were observed.

## Discussion

Clinical features and biomarkers have limitations in the early detection of HF, and multiple studies have found LUS—even in non-expert hands—to be significantly more predictive. Other accurate remote monitoring technologies such as the CardioMEMS device are expensive and invasive. Thus, LUS, an easy to use, non-invasive, and affordable technology, is an appealing and promising alternative.

This small study addresses the feasibility of patient-performed remote LUS, with image transmission for clinician interpretation, for the early detection of HF. While the sample size is small, we found that patient-performed remote LUS is feasible, and that it can result in a high percentage of interpretable clips that can be used to identify increasing extravascular lung water—an early harbinger of a HF exacerbation.

Very few studies have evaluated the use of patient-performed ultrasound in general. Only one recent study [24] was published looking specifically at patient-performed LUS in HF patients. In this study, participants were shown a 15 min training video on a four-point LUS self-exam and then asked to perform the same exam without assistance. While this study also resulted in a high degree of interpretable clips, the four-point exam did not include the mid-axillary line, which has the highest diagnostic accuracy for HF. In addition, each participant only performed the LUS once, in the emergency department of a hospital, immediately after watching a training video. This does not accurately simulate use of LUS at home on an on-going basis, as it would need to be used clinically to detect HF in outpatients.

Our study demonstrates that patient-performed remote LUS is not only feasible, it is also relatively quick to perform, with an average ultrasound time of 5.04 min. While this time does not include the patient set up prior to obtaining clips, that set up time is relatively minimal and can be done before a provider joins the video call. A medical video visit of 15 to 20 min is comparable in length to other clinic visits, and it would be a reasonable replacement for an in-person HF follow-up appointment.

The average dry weight for patients in this study was 106.3 kg, and yet that did not compromise the quality of the LUS clips. This is an important finding, given that many patients with HF are overweight.

Finally, the two patients who continued performing LUS without supervision after the conclusion of their study participation transmitted clips that were 100% interpretable. This suggests that patients may even be able to perform LUS without the need for provider presence after initial training and supervision. This would potentially enable longer term, ongoing remote monitoring of HF patients with many fewer clinic visits.

While these initial findings are very encouraging in terms of the feasibility of tele-guided patient-performed LUS, no HF decompensations were observed amongst these first 8 patients. To adequately determine whether the technology can be used to detect early decompensations, more patients need to be included in the study and the study period needs to be extended to witness decompensations.

In addition, the results of our study may not be generalizable to the typical heart failure population. Our patients were technologically savvy and relatively young, and may not be representative of the general heart failure population. More elderly patients with less experience with technology may not be suitable for this technology. However, this statement is not generalizable, as more patients of advanced age are becoming familiar with the use of smartphones. The study will need to be expanded to a much larger number to better define the group of heart failure patients suitable for this technology. Other issues that need to be investigated in future studies include cost effectiveness and the place of this new technology in the current clinical management landscape.

## Conclusions

Patient-performed remote LUS in HF patients is feasible, with over 90% of exams being diagnostically valid, with at least 4 out of 6 interpretable clips. No HF exacerbations were observed in this study. Thus, to determine whether remote LUS can be used to detect early HF decompensations, the study period needs to be extended and more patients need to be included.

## Data Availability

The data sets used and/or analyzed during the current study are available from the corresponding author on reasonable request.

## Abbreviations

HF: Heart failure
LUS: Lung ultrasound
EF: Ejection fraction
ICD: Implantable cardioverter defibrillator
PAP: Pulmonary artery pressure
FDA: Food and Drug Administration
NYHA: New York Heart Association
